# Ionizing radiation exposure during adulthood and risk of developing central nervous system tumors: systematic review and meta-analysis

**DOI:** 10.1038/s41598-022-20462-7

**Published:** 2022-09-28

**Authors:** Julie Lopes, Clémence Baudin, Klervi Leuraud, Dmitry Klokov, Marie-Odile Bernier

**Affiliations:** 1 Laboratory of Epidemiology (LEPID) – Institute for Radiological Protection and Nuclear Safety (IRSN), 92262 Fontenay-aux-Roses, France; 2 Laboratory of Radiobiology and Radiotoxicology (LRTOX) – Institute for Radiological Protection and Nuclear Safety (IRSN), 92262 Fontenay-aux-Roses, France

**Keywords:** Neurological disorders, CNS cancer, Epidemiology

## Abstract

Many studies on ionizing radiation (IR) exposure during childhood have shown deleterious effects on the central nervous system (CNS), however results regarding adult exposure are inconsistent, and no systematic reviews have been performed. The objectives are to synthesize the findings and draw evidence-based conclusions from epidemiological studies on the risk of benign and malignant brain and CNS tumors in humans exposed to low-to-moderate doses (< 0.5 Gy) of IR during adulthood/young adulthood. A systematic literature search of four electronic databases, supplemented by a hand search, was performed to retrieve relevant epidemiological studies published from 2000 to 2022. Pooled excess relative risk (ERR_pooled_) was estimated using a random effect model. Eighteen publications were included in the systematic review and twelve out of them were included in a meta-analysis. The following IR sources were considered: atomic bombs, occupational, and environmental exposures. No significant dose-risk association was found for brain/CNS tumors (ERR_pooled_ at 100 mGy = − 0.01; 95% CI: − 0.05, 0.04). Our systematic review and meta-analysis did not show any association between exposure to low-to-moderate doses of IR and risk of CNS tumors. Further studies with histological information and precise dose assessment are needed.

## Introduction

Exposure to ionizing radiation (IR) is ubiquitous and can be anthropogenic (medical procedures, releases from nuclear facilities, nuclear accidents, or nuclear weapons tests) or natural (radon, telluric and cosmic rays, radionuclides in soils). The United Nations Scientific Committee on the Effects of Atomic Radiation (UNSCEAR) has estimated the global average annual effective dose of about 3.0 millisievert (mSv) per person, including 2.4 mSv from natural sources and 0.6 mSv from artificial sources^1^. While most of the population is exposed to relatively low levels of background IR, some people may be additionally exposed to IR due to their lifestyle (e.g., frequent flyers), occupation, health condition (diagnostic or therapeutic medical exposure), or environment (e.g., high background radiation areas). Many epidemiological studies have demonstrated that exposure to IR can increase the risk of cancer for some specific sites^2^, including central nervous system (CNS)^1^.

Exposure to moderate-to-high-dose of IR (> 0.5 Gy) during childhood is an established risk factor for CNS cancers (all brain/CNS tumors, gliomas and meningiomas)^2,3^, as reported in studies of survivors of pediatric primary tumors^4^ and in children irradiated for benign medical conditions^5,6^. At lower doses (< 0.1 Gy), such as those received during computerized tomography (CT) scans, some studies reported a dose–response relationship between exposure to IR and CNS tumors^7–9^. A summary of these studies completed by a meta-analysis concluded to a positive dose–response relationship^10^. Regarding exposure to medical diagnostic X-ray in utero, the relationship with brain cancer incidence was significant in the Oxford Survey, and only marginally non-significant for the combined other studies^11^. However, caution should be exercised when evaluating diagnostic medical exposures as study results have been shown to be subject to significant uncertainties and biases (e.g., poorly documented historical exposure data, limited non-target organ dosimetry, bias by indication, etc.)^12^.

The extrapolation of results from pediatric patients to adults should be considered with caution since for a given radiation dose, children are generally at greater risk of tumor than adults, because of their higher radiosensitivity^13^. Moreover, the consequences of IR low-dose exposures regarding adulthood on CNS tumors risk are less clearly demonstrated than in children. The Life Span Study of atomic bomb survivors exposed to moderate radiation during adulthood found no significant association with brain cancer occurrence^14^, whereas a significant dose-dependent excess of schwannoma for those exposed from age 40 onwards had been previously observed^15^. Because of the increase of the use of IR in the workplace since the 1950s, workers (e.g., nuclear and medical workers, aircrew) are prone to be exposed to low-to-moderate dose range (< 0.5 Gy) of IR but received at low-dose rate. Accordingly, the effect of adult exposure to IR on the risk of CNS tumors have been studied in several epidemiological studies in the recent years. However, results of those studies are inconsistent, with some reporting no association between IR exposure and malignant or benign CNS tumors risk^16–18^. However, some cases reports raised concerns about potential higher incidences of brain tumor in healthcare professionals^19,20^.

Thus, the objectives of the present systematic review are to (1) identify pertinent studies, synthesize their results, and draw evidence-based conclusions from epidemiological studies carried out on the risk of malignant and benign brain and CNS tumors (including malignant neoplasm of spinal cord, cranial nerves and other parts of the nervous system) in people exposed to low-to-moderate doses of IR (< 0.5 Gy) during adolescents above 16 years old and during adulthood, and (2) to provide a quantitative summary of the overall risk estimate.

## Methods

The Preferred Reporting Items for Systematic Reviews and Meta-Analyses (PRISMA) guidelines were used to guide our literature review and synthesis (supplementary material, Table S1)^21^. The protocol was recorded in the PROSPERO database (Registration Number: CR42021215479).

### Data source and search

This work is part of a larger research on tumoral and non-tumoral^22^ effects in the brain/CNS system following exposure to IR in young adulthood and adulthood. This study considers only the effects relevant to tumor development.

An online-based literature search was conducted in May 2022 in PubMed, Scopus, Web of Science, and Google Scholar databases. The first query included a combination of outcome, exposure, and population keywords: (neuro* OR nervous OR brain OR brain cancer OR cerebro*) AND (ionizing radiation OR medical radiation OR cosmic radiation OR nuclear OR radon OR background radiation) AND (patient* OR human OR worker OR cohort OR epidemiolog*). To complete this query, additional queries were performed to catch studies with no keywords in the title nor in the abstract: cosmic radiation AND mortality OR incidence; (nuclear worker OR nuclear facility OR nuclear industry) AND mortality OR incidence; ionizing radiation AND mortality OR incidence. Also, additional articles were searched from the references cited by relevant publications and international reports^1,2^. Duplicates from the different databases were removed.

For the selection process, we proceeded as follows: (1) the articles obtained through the queries were selected on the title; (2) the abstracts of the selected articles were read, and a further selection was performed; (3) the articles were selected on the full-text screening. The selection was carried out by two independent investigators (J.L. and C.B.), whereas a third investigator (M.-O.B.) made a decision in case of disagreement.

### Inclusion and exclusion criteria

Eligible studies were cohort, case–control, and cross-sectional studies, published in English between January 2000 and May 2022. The publication period criterion allows for the inclusion of studies whose radiation exposure is more similar to the current IR exposures, especially in medical professionals, given improved radiation protection regulations and decreasing doses^23^. Furthermore, older good quality studies are regularly updated and would be found as their last updated publication. Last, it allows the exclusion of some ancient studies with poor quality design and ensure uniformity of studies in their structure (introduction, methods, results, discussion, conclusions) and the way they were reviewed (homogeneous judgment criteria), thus facilitating the comparison of studies between them. All the studies concerned exposures to low-to-moderate doses of IR (< 0.5 Gy), during adulthood or adolescence (at least 16 years old) as companies involved in some of the studies included in this work allowed for work at age 16 or older. To be eligible, a study had to report on incidence or on mortality from CNS tumors coded according to the International Classification of Diseases (ICD), either its revision 9 (ICD-9: 191-192)^24^ or 10 (ICD-10: C70-C72, D32-D33, D42-D43)^25^. Because most studies of ICD-9 codes 191-192 have reported results for both codes together, non-cranial tumors (i.e., code 192) were also included in the present work. The definitions of the ICD codes are provided in supplementary data (Table S2).

Exclusion criteria were the followings: studies with less than 10 cases; conference abstracts, reports, meta-analyses, letters, and ecological studies; all the studies with no dose assessment, or in which exposure assessment was only based on self-reports or questions about IR exposure (e.g., “How many dental X-rays have you been exposed to in your lifetime?”). However, the references of these excluded studies were checked to retrieve potential studies that met the inclusion criteria of the present review. In case of publications on overlapping populations or study updates, only data from the most complete study were considered. Studies with quantitative estimates were included in the meta-analysis section. Meta-analysis was performed when at least three studies were eligible.

### Quality assessment of individual studies

The quality of the included studies was assessed using the Newcastle Ottawa Scale (NOS) for quality assessment of epidemiological studies^26^, which is usually used in systematic review process. This evaluation is based on eight items, which are categorized into three groups: selection of study groups, comparability of groups, and outcome of interest, for cohort studies. Stars are attributed for each item depending on the quality and a score (0 to 9) is obtained by adding the stars of each item. A study with an average NOS score of at least 6 stars out of 9 is considered as good quality.

### Statistical analysis

Details on sample size, number of exposed/unexposed people, total cases or deaths, information about gender, age at exposure, and estimates of measures of risk such as excess relative risk (ERR), relative risk (RR), hazard ratio (HR), standardized rate ratio (SRR), proportional incidence ratio (PIR), or incidence rate ratio (IRR) were collected for each study when available. A pooled ERR was estimated to assess the strength of the association when available from the individual studies, using an alternative of the DerSimonian and Lair-based model proposed by Richardson et al.^27^. This method uses a parametric transformation of published results to improve the normal approximation used to estimate confidence intervals. This approach provides less biased summary estimates than the traditional meta-analysis approach, with more precise confidence interval coverage.

Heterogeneity across studies was tested using Cochran’s Q test at *p* < 0.1 and quantified using the I^2^ statistics. The latter reflects the proportion of total variance estimated to be attributable to between-study heterogeneity. Heterogeneity was considered as null, low, moderate, and high for I^2^ values < 25, 25–50, 50–75, and > 75%, respectively. Publication and selection biases were assessed and tested using the Egger test. Statistical significance was defined by *p* < 0.05.

Meta-analyses were based on the original values reported by the studies. Whereas a study reported only a 90% (as opposed to 95%) confidence interval^18^, this was introduced in the main meta-analyses, and a sensitivity analysis was performed with a 95% confidence interval calculated by ourselves.

Statistical analyses were conducted with the R 3.6.3 software (R Foundation for Statistical Computing, Vienna, Austria) using the metafor and Metaan packages.

## Results

From 11,485 articles retrieved, 2,063 were excluded as being duplicates and 9,422 articles were screened on title; next, the abstract of 528 of them were reviewed. Finally, 201 articles were full read, of which 17 were selected. Briefly, full texts were excluded because of overlaps (62 studies), outcome not in the scope of the review (48 studies), unsuitable exposure (46 studies), study design not meeting inclusion criteria (20 studies), or for other reasons (8 studies, for combination of several exclusion criteria).

**Figure 1:**
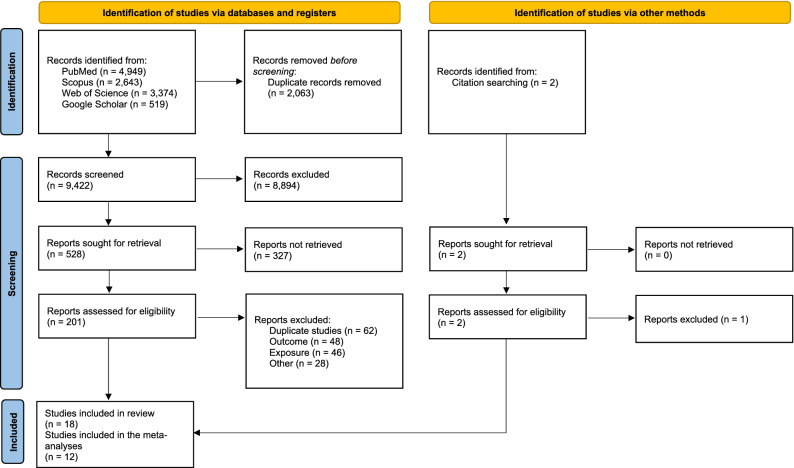
Preferred Reporting Items for Systematic Reviews and Meta-Analyses 2020 flow diagram for new systematic reviews which included searches of databases, registers and other sources.

One more article was obtained through search in bibliographic references as described above, for a total of 18 articles included in this systematic review. Of those, 12 presented quantitative results that were included in the meta-analyses (Fig. 1). The characteristics, key findings, and NOS score assessments of the 18 articles included in the present review are detailed in Table 1. Each of them was based on cohorts of IR exposed people, and the majority was published after the year 2010. Most studies investigated CNS tumors risk in relation to occupational exposures (16 studies), while others addressed environmental background exposure or atomic bomb exposure (2 studies).

**Table 1:** Characteristics and key findings of the included studies on brain/CNS tumors.

First author, year	Country	Population	Design	Exposure assessment	Outcome(s)	Major results	NOS scores
**Nuclear workers and uranium miners**							
Boice et al. 2021^28^	USA	19,808 (M), 6520 (F) workers at the Los Alamos National Laboratory	Cohort	Brain radiation absorbed dose, combining external and internal sources for Pu: mean: 11.6 mGy, median: 0.76 mGy, max: 760 mGy	Brain, central nervous system cancer (ICD-9: 191-192)	HR (95% CI) at 100 mGy: 1.24 (0.78, 1.98), n_deaths_ = 94 ERR (95% CI) at 100 mGy: 0.20 (− 0.27, 0.67), n_deaths_ = 94	8
Golden et al. 2019^32^	USA	2514 (M) Mallinckrodt uranium processing workers	Cohort	Brain dose from all sources of external and internal radiation combined: mean: 37.2 mGy, max: 750 mGy	Brain, central nervous system cancer (ICD-9: 191-192)	HR (95% CI) at 100 mGy: 0.78 (0.29, 2.10), n_deaths_ = 22 ERR (95% CI) at 100 mGy: − 0.13 (− 0.55, 0.29), n_deaths_ = 22	8
Richardson et al. 2018^18^	France, UK, USA	268,262 (M), 40,035 (F) nuclear workers (INWORK)	Pooled cohort	M: mean cumulative dose to the brain: 20.2 mGy, median (IQR): 4.3 (0.9, 17.5). 95th percentile: 94.2 mGy F: mean cumulative dose to the brain: 4.3 mGy, median (IQR): 1.1 (0.4, 3.6). 95th percentile: 17.1 mGy	Brain, central nervous system cancer (ICD-9: 191-192)	ERR (90% CI) per Gy: − 0.92 (< − 0.92, 1.14), n_deaths_ = 594	8
Rage et al. 2017^33^	France	5400 (M) uranium miners	Cohort	Cumulative exposure (WLM), mean (se): 35.1 (69.9), median (min–max): 10.8 (0.002–960.1)	Brain, central nervous system tumor (ICD-10: C70-C72, D32-D33, D42-D43)	ERR (95% CI) per 100 WLM: − 0.12 (NA, NA), n_deaths_ = 28	7
Sokolnikov et al. 2015^30^	Russia	19,395 (M), 6362 (F) Mayak workers	Cohort	Mean external cumulative colon dose: 354 mGy	Brain, central nervous system cancer (ICD: NA)	ERR (95% CI) per Gy: < 0.00 (< − 0.10, 0.32), n_deaths_ = 66	8
Zablotska et al. 2014^31^	Canada	37,697 (M), 7619 (F) nuclear workers	Cohort	Mean person-time weighted total dose lagged by 10 years (range, SD), mSv: 21.6 (0.0–678.8, 47.0)	Brain, central nervous system cancer (ICD: NA)	ERR (95% CI) per Sv: − 1.45 (< − 1.47, 5.83), n_deaths_ = 22	8
Howe et al. 2004^29^	USA	47,311 (M), 6387 (F) nuclear power industry workers	Cohort	Mean cumulative equivalent dose: 28.5 mSv (M), 4.6 mSv (F) and 25.7 mSv for the all cohort	Brain, central nervous system cancer (ICD: NA)	RR (95% CI) by cumulative dose categories: < 1 mSv: 1.00 (ref); 1–49 mSv: 0.60 (0.20, 1.78); 50- mSv: 0.41 (0.05, 3.74) ERR (95% CI) per Sv = − 2.50 (< − 2.51, 27.1), n_deaths_ = 23	8
**Medical workers**							
Boice et al. 2021^28^	USA	55,218 (M), 53,801 (F) medical and associated radiation workers	Cohort	Mean cumulative absorbed dose to the brain: 18.9 mGy (max: 1.08 Gy)	Brain, central nervous system cancer (ICD-9: 191, 192.0–192.1)	HR (95% CI) at 100 mGy: 1.23 (0.74, 2.03), n_deaths_ = 165 ERR (95% CI) at 100 mGy: 0.20 (− 0.30, 0.71), n_deaths_ = 165	8
Lee et al. 2021^36^	South Korea	53,582 (M), 40,338 (F) diagnostic medical radiation workers	Cohort	Mean cumulative badge dose: 7.20 mSv (IQR 0.21–5.41 mSv)	Brain, central nervous system cancer (ICD-10: C70-C72)	RR (95% CI): < 1 mSv: 1.00 (ref); 1–5 mSv: 0.48 (0.19, 1.20); 5–20 mSv: 1.07 (0.51, 2.24); ≥ 20 mSv: 0.66 (0.26, 1.63) (M/F) ERR (95% CI) per 100 mGy: − 0.29 (− 3.14, 2.55), n_deaths_ = 43 (M/F)	8
Kitahara et al. 2017^17^	USA	26,642 (M), 83,655 (F) radiologic technologists	Cohort	Cumulative mean absorbed brain dose: 12 mGy (range, 0–290 mGy)	Malignant intracranial neoplasm of the brain and central nervous system (ICD-9: 191,192.0, 192.1 / ICD-10: C70.0, C70.9, C71, C72.2-C72.9)	ERR (95% CI) per 100 mGy: 0.10 (< − 0.30, 1.50), n_deaths_ = 193 (M/F) ERR (95% CI) per 100 mGy: 0.90 (< − 0.30, 4.60), n_deaths_ = 64 (M) ERR (95% CI) per 100 mGy: − 0.30 (< 0.00, 1.00), n_deaths_ = 129 (F)	9
**Flight attendants**							
Dreger et al. 2020^35^	Germany	6006 (M) cockpit crew, 17,017 (F) cabin crew	Cohort	Collective cumulative effective doses (in mSv): median: 44.1 (IQR: 30.5–54.1, max: 99.7) and 25.1 (IQR: 10.5–46.6, max: 96.7) for male cockpit and female cabin crew respectively	Brain, central nervous system cancer (ICD: NA)	RR (95% CI) per 10 mSv: 1.03 (0.76, 1.45) (M) RR (95% CI) per 10 mSv: 0.83 (0.48, 1.31) (F)	8
Yong et al. 2014^34^	USA	5958 (M), 6 (F) cockpit crew	Cohort	Mean annual cosmic radiation dose: 1.4 mSv (median: 1.4 mSv, range: 0.0042–2.8 mSv)	Brain, central nervous system cancer (ICD: NA)	SRR (95% CI) per cumulative radiation dose: No lag: 0– < 22.9 mSv: ref; 22.9– < 35.1 mSv: 0.84 (0.27, 2.63); 35.1– < 44.8 mSv: 1.50 (0.56, 4.04); 44.8 + mSv: 1.27 (0.39, 4.10) 10-year lag: 0– < 18.1 mSv: ref; 18.1– < 32.5 mSv: 1.29 (0.41, 4.00); 32.5– < 43.7 mSv: 1.13 (0.35, 3.68), 43.7 + : 3.84 (1.00, 14.74) HR (95% CI) cumulative radiation dose per 10 mSv: unlagged: 2.17 (1.06, 4.81); 5-year lag: 2.37 (1.09, 5.61); 10-year lag: 2.37 (1.01, 6.12)	8
**Atomic bomb survivors**							
Brenner et al. 2020^14^	Japan	1,176,020 PY (M/F) Life Span Study of atomic bomb survivors	Cohort	Radiation dose estimates to the brain contributing 3.1 million person-years of observation. Mean dose of the cohort (range): 0.13 Gy (0 to 3.8 Gy)	Death and incidence by glioma, meningioma, schwannoma. Other/NOS (ICD-O-3T): C70.0, C70.1, C70.9, C71.0-C71.9, C72.0-C72.5, C72.8-C72.9, C75.1-C75.3	Age at exposure: 20 + . ERR per Gy (95% CI): All CNS: 1.10 (− 0.02, 2.97); Glioma: 1.70 (< − 0.73, 7.83); Meningioma: 2.24 (< − 0.17, 7.11); Schwannoma: − 0.06 (< − 1.70, 3.55)	9
**Military using nuclear materials**							
Gillies et al. 2022^40^	UK	21,357 (M) UK participants in the UK's atmospheric nuclear weapons tests and experimental programs compared to a group of 22,312 (M) controls	Cohort	8% of the total participants cohort had non-zero recorded radiation doses (mean dose from gamma radiation: 9.9 mSv)	1 – Brain, central nervous system tumor (ICD-9: 191-192, 225 / ICD-10: C70-C72, D32-D33) 2 – Benign brain and central nervous system tumor (ICD-9: 225 / ICD-10: D32-D33)	1 – Death: RR (90% CI): 0.99 (0.80, 1.22); Incidence: RR (90% CI): 1.05 (0.87, 1.27) 2 – Incidence: RR (90% CI): 1.97 (1.29, 3.02)	8
Friedman-Jimenez et al. 2022^38^	USA	85,033 (M) who had served on a nuclear-powered submarine in the US Navy	Cohort	Mean and median cumulative radiation doses: 5.7 and 1.1 mSv, range 0–242 mSv	Brain, central nervous system cancer (ICD: NA)	ERR (95% CI) per 10 mSv: 0.025 (− 0.33, 0.38), n_deaths_ = 43	8
Boice et al. 2020^39^	USA	114,270 (M) military participants at eight aboveground nuclear weapons test series	Cohort	Gamma radiation dose: mean: 6 mSv, max: 908 mSv	Brain, central nervous system cancer (ICD-9: 191-192)	HR at 100 mGy (95% CI): 0.25 (0.08, 0.73), n_deaths_ = 495 ERR at 100 mGy (95% CI): − 1.40 (− 2.50, − 0.32) n_deaths_ = 495	7
**Chernobyl cleanup workers**							
Rahu et al. 2013^41^	Estonia, Latvia, and Lithuania	17,040 (M) Chernobyl cleanup workers	Cohort	External whole-body radiation dose: average dose: 10.9 cGy (9.9 cGy, 11.8 cGy and 10.9 cGy in Estonian, Latvian, and Lithuanian sub-cohorts respectively) and interquartile range of 5.2–16.3 cGy	1 – Brain, central nervous system cancer (ICD-10: C70-C72) 2 – Brain cancer (ICD-10: C71)	1 – PIR (95% CI): 1.24 (0.85, 1.75), n_diseases_ = 32 2 – PIR (95% CI): 1.16 (0.77, 1.68), n_diseases_ = 28	8
**Environmental radiation**							
Bräuner et al. 2013^42^	Denmark	24,533 (M), 27,141 (F) Danish exposed to residential radon	Cohort	Median estimated radon: 40.5 Bq/m^3^	Incidence of benign and malignant brain tumor (ICD-10: C71, D33.0-D33.2 and D43.0-D43.2)	IRR (95% CI) per 100 Bq/m^3^: 1.96 (1.07, 3.58), n_diseases_ = 121	8

*M* Male; *F* Female; *Gy* Gray; *Sv* Sievert; *ICD* International Classification of Diseases; *ICD-O* International Classification of Diseases for Oncology; *RR* Relatif Risk; *HR* Hazard ratios; *ERR* Excess Relatif Risk; *SRR* Standardized Risk Ratio; *PIR* Proportional Incidence Ratio; *NA* Not Available; *IQR* Interquartile Range; *IRR* Incidence Risk Ratio; *NOS* Not Otherwise; Specified *NOS scores* scores obtained through the Newcastle Ottawa Scale.

### Occupational worker studies

#### Nuclear workers and uranium miners

Five articles were focused on nuclear workers^18,28–31^. In a cohort of 26,328 Los Alamos National Laboratory workers exposed to a combination of photons, neutrons, tritium and plutonium (among which 17,053 workers were monitored for a combination of external and internal source for plutonium and had dose information available; cumulative brain radiation absorbed dose: mean: 11.6 mGy; max: 760 mGy), Boice et al. (2021) reported a non-significant positive dose–response relationship among the whole cohort (ERR at 100 mGy: 0.20; 95% CI: − 0.27, 0.67; n_deaths_ = 94)^28^. Similarly, Richardson et al. 2018 reported a non-significant negative dose–response relationship between IR (median cumulative brain dose of 20.2 mSv) and brain/CNS cancers in the international pooled study of radiation workers from the UK, the USA and France including 308,297 workers (ERR = − 0.92; 90% CI: <  − 0.92, 1.14; n_deaths_ = 594) (INWORKS cohort)^18^. Likewise, Sokolnikov et al. observed a non-significant negative dose–response relationship between occupational IR exposure (mean external brain dose: not available) and brain cancer among the cohort of Mayak Production Association workers in Russia first employed in 1948–1982 and followed up until 2008 (ERR per Gy =  < 0.00; 95% CI: <  − 0.10, 0.32; n_deaths_ = 66)^30^. Zablotska et al. also found a non-significant negative dose–response relationship between IR exposure (cumulative person-time weighted lung dose: 21.64 mSv, max: 678.78 mSv) and brain/CNS cancers among a cohort of 45,316 Canadian nuclear workers (ERR per Sv = − 1.45; 95% CI: <  − 1.47, 5.83; n_deaths_ = 22)^31^. Furthermore, non-significant negative dose–response relationship was reported among 53,698 US nuclear power plant industry workers between IR exposure (mean cumulative dose: 25.7 mSv) and brain/CNS cancers (ERR per Sv = − 2.50; 95% CI: < − 2.51, 27.1; n_deaths_ = 23)^29^.

Similar results were found in uranium miners and processing worker studies: non-significant negative dose–response relationship was found among 2,514 Mallinckrodt uranium processing workers exposed to a combination of X-ray, uranium and radium (mean brain dose from all sources of external and internal radiation combined: 37.2 mGy; max: 750 mGy) and brain/CNS cancers (ERR at 100 mGy = − 0.13; 95% CI: − 0.55, 0.29; n_deaths_ = 22)^32^; a non-significant negative dose–response relationship was reported between radon exposure (cumulative radon exposure: 35.1 WLM) and brain/CNS cancers among 5,400 French uranium miners followed up from 1946 to 2007 (ERR per 100 WLM = − 0.12; 95% CI: NA, NA; n_deaths_ = 28)^33^.

#### Flight attendants

Yong et al. reported a significant relationship between cumulative dose of galactic cosmic radiation (mean: 28 mSv, max: 71 mSv) and brain/CNS cancers mortality among 5,964 former US commercial cockpit crew (pilots and engineers) (HR per 10 mSv = 2.37; 95% CI: 1.01, 6.12; n_deaths_ = 32 under the hypothesis of a 10-year lag in cumulative dose)^34^. Whereas, Dreger et al. found no evidence of a dose–response relationship between cumulative effective dose (overall median: 34.2 mSv, max: 116 mSv) and brain/CNS cancers risk among a cohort of 26,846 aircrew personnel^35^.

#### Medical workers

No statistically significant association was reported between cumulative radiation exposure (mean badge dose: 7.2 mSv, max: >20 mSv) and brain/CNS cancers among diagnostic medical radiation workers in South Korea (ERR per 100 mGy = − 0.29; 95% CI: − 3.14, 2.55; n_deaths_ = 43)^36^. Similarly, no significant association was observed between cumulative occupational radiation exposure to the brain (cumulative mean absorbed brain dose: 12 mGy, max: 290 mGy) and mortality from malignant brain/CNS cancers in the US Radiologic Technologists (ERR per 100 mGy: 0.1; 95% CI: <  − 0.30, 1.50; n_deaths_ = 193)^17^. Finally, the recent study of medical radiation workers in the United States found that cumulative absorbed dose to the brain (mean: 18.9 mGy, max: 1.08 Gy) was not significantly associated with brain cancers (ERR at 100 mGy = 0.20; 95% CI: − 0.30, 0.71; n_deaths_ = 165)^37^.

#### Military using nuclear materials

Friedman-Jimenez et al. found a non-significant dose–response relationship between IR exposure (mean cumulative IR badge dose: 5.7 mSv, max: 242 mSv) and brain/CNS cancers (ERR per 10 mSv = 0.025; 95% CI: − 0.33, 0.39; n_deaths_ = 43) among 85,033 enlisted men who had served on nuclear-powered submarines in the United States Navy between 1969 and 1982^38^. Boice et al. found a significant negative association between radiation dose (mean NuTRUS film badge gamma radiation dose: 6 mSv, max: 908 mSv) and brain/CNS cancers (ERR at 100 mGy = − 1.40; 95% CI: − 2.50, − 0.32; n_deaths_ = 495) among US military participants to atmospheric tests in Nevada and Pacific from 1945 to 1962^39^. Gillies et al. found similar mortality and incidence relative risks in 21,357 males UK participants followed between 1952 and 2017 in the UK’s atmospheric nuclear weapons tests and experimental programs, of whom 8% had non-zero recorded radiation dose from gamma radiation (mean: 9.9 mSv), compared to a group of 22,312 males controls (RR = 0.99; 95% CI: 0.80, 1.22 and RR = 1.05; 95% CI: 0.87, 1.27 respectively)^40^.

#### Chernobyl cleanup workers

A significantly increased incidence of malignant brain tumors was observed in clean-up workers from the Baltic countries who worked in the Chernobyl area in 1986 and stayed onsite for more than 90 days, in comparison with the males of the general population of each country (PIR = 2.08; 95% CI: 1.07, 3.63; n_deaths_ = 12), but there was no trend when PIR were given by dose category^41^. However, the accuracy of the diagnosis and the representativeness of the unexposed cohort are an issue in this study.

### Atomic bombing and environmental studies

#### Atomic bombing survivors

While a significant linear dose–response relationship was found for all CNS tumors (glioma, meningioma, schwannoma, and other/not otherwise specified tumors) in the entire population of the LSS study of atomic bomb survivors from Hiroshima and Nagasaki, the ERR at 1 Gy was no longer statistically significant when age at exposure was restricted to the age of 20 and more (ERR per Gy = 1.10; 95% CI: − 0.02, 2.97; n_deaths and diseases_ = 92 in the group > 25 years old at exposure)^14^.

#### Environmental radiation

A statistically significant association between residential radon and brain tumors (including benign) incidence (IRR = 1.96; 95% CI: 1.07, 3.58; n_diseases_ = 121), accounting for a 10-year latency period, was shown in a Danish cohort comprising 57,053 individuals recruited between 1993 and 1997 exposed to a median estimated radon dose of 40.5 Bq/m^3^^42^.

#### Meta-analyses

**Figure 2:**
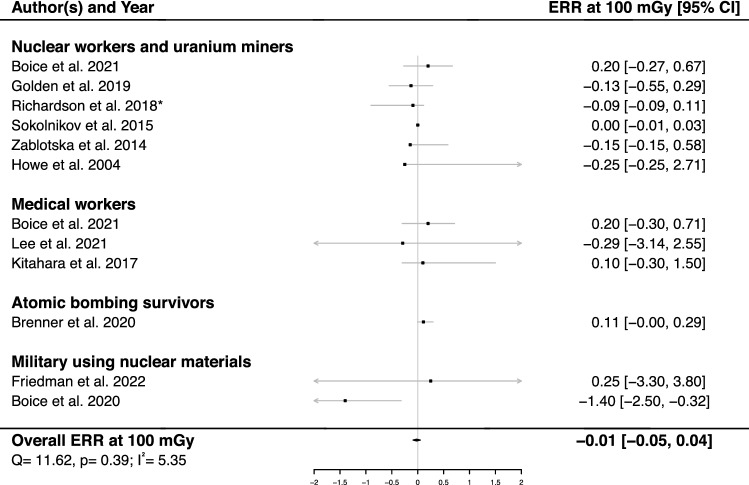
Excess relative risk (ERR) and 95% confidence interval (CI) for brain/CNS tumor death in relation to IR-exposure. *90% CI. We calculated an ERR per Gy with a 95% CI for the Richardson study and this does not change the outcome of this meta-analysis.

A pooled ERR at 100 mGy was calculated using 12 studies^14,17,18,28–32,36–39^, showing no dose-risk association (ERR_pooled_ = − 0.01; 95% CI: − 0.05, 0.04) and no heterogeneity (Q = 11.62, *p* = 0.39 and I^2^ = 5.35%), nor publication bias (*p* = 0.56). (Fig. 2).

Supplementary analyses were carried out for mortality and incidence rates in cohorts compared to those in the general populations (supplementary material, Table S3).

## Discussion

Brain/CNS tumors risks after adult and adolescent older than 16 years exposure to low-to-moderate doses of IR was analyzed based on 18 studies. Our results suggest no dose–response relationship between IR exposure and brain/CNS tumors death using ERR.

Our results contrast with the previously increased risk of CNS tumors after childhood exposure to low-to-high doses of radiation, as reported in the UNSCEAR 2006 Report^2^, and more specifically in studies of survivors of pediatric primary tumors^4^ and in children irradiated for benign medical conditions^5,6^. Since children have a higher radiosensitivity, this difference in results does not appear surprising because of the focus on adulthood exposure in our study. The rate of cell proliferation during neurogenesis in the adult brain is much lower compared to that in the developing brain of children and to other adult tissues, such as bone marrow, intestine and lung^43^. Consequently, brain cancer in adults accounts only for a small fraction of all cancers (~ 1%), whereas in children, brain tumors are the most common solid tumors^44^. It is possible that the association between IR exposure and CNS tumors may mostly be driven by the pediatric subjects. Overall, it seems that although our results reveal a lack of correlation between exposure to low-to-moderate doses of IR in adults and the risk of CNS tumors incidence and mortality (corroborated by comparisons of mortality and incidence rates with those of the general population, Figs. S1 and S2), there are still many factors that need to be considered in future studies.

Among them, several environmental risk factors are currently studied and debated: non-ionizing radiation (e.g., radiofrequency, electromagnetic fields)^45^, pesticides, heavy metals (e.g., lead, mercury), nitro compounds, certain viral infections (e.g., SV40) and cigarette smoking^46–48^. Other factors that affect the incidence of brain cancer include previous history of allergy (reducing the risk)^49^, regular use of certain medication^50^, diet and lifestyle^51,52^ and others. Accounting for these factors in IR-exposed populations would help delineate the link between IR and the risk of brain tumors. Furthermore, exposure to IR in the occupational setting is often accompanied by co-exposure to other health risk factors (e.g., chemical substances, pesticides, heavy metals, nitro compounds, non-ionizing radiations, air pollution, tobacco use, etc.) and may confound and/or modify the relationship between IR exposure and a health outcome. While research on health effects of co-exposures to two or more risk factors (exposome) is a very dynamic area of research and there are examples of synergies or antagonisms following co-exposure to different environmental agents^53,54^, overall, the interaction of various factors and associated health outcomes are poorly characterized to date. It is also estimated that 5% of brain tumors are related to hereditary factors^55^, with even smaller percentage in adult brain cancers patients.

The most informative way to assess causality is to estimate dose-risk relationships. Accordingly, studies which presented only SMRs or SIRs were included only in supplementary material for information. Of the 18 studies included in this work, 12 reported dose–response relationships and were included in the meta-analysis. The pooled ERR was non-significant, leading to the conclusion that there was no association between adult IR exposure and brain/CNS tumors risk. However, uncertainties in dosimetry assessment can obscure the relationship. We can note a lack of accurate dosimetry reconstruction for several studies, as those focusing on medical workers, and encourage future research to improve dose assessment in studies, including as much as possible homogenization of the dosimetric units used. Indeed, nine out of the twelve studies with dose–response analyses considered absorbed doses to the brain (in Gy)^14,17,18,28,30,32,36,37,39^, whereas the three other studies^29,31,38^ used equivalent whole-body doses (in Sv) from external photons based on individual monitoring records . However, we calculated a pooled ERR/Gy using the numerical values of the estimated ERR/Sv as they are reported in the studies, assuming that the brain dose from external photons correlates with the whole-body equivalent dose, yet being aware that the absorbed dose to the brain is certainly lower than the equivalent whole-body dose^18,56^.

Among the studies included in the meta-analysis, conditions of exposure to IR were heterogeneous: uranium miners are repeatedly exposed internally and externally to a mix of radon gas and its progenies, external gamma rays, and uranium dust^57^; nuclear workers are predominantly exposed to external gamma rays, possibly combined with tritium, uranium, plutonium, or neutrons depending on their activity^30,56^; medical radiation workers are predominantly exposed to X-rays^58^; the atomic bomb survivors were acutely exposed to external irradiation^59^. Some inconsistency in the results of the included studies may be explained by the different conditions of radiation exposure. In the meta-analysis, four out of twelve studies focused on radiation workers or uranium miners possibly exposed to internal contamination by radionuclides^18^ such as uranium or plutonium^30,32^ or by tritium^31^, whereas only the external exposure has been considered, except for the study on Mallinckrodt uranium processing workers^32^. The dose to the brain due to radionuclides intakes was not taken into account in the dose-risk analyses but this should not induce a significant bias because only a small proportion of workers are concerned. Although it is generally assumed that radionuclides may deposit only in small proportion in the brain leading to a possibly limited impact on CNS tumors risk, it is currently suspected that improvements in dose estimation for internal emitters are needed to better characterize their impact on the brain^60^.

A common limit of the considered studies is the lack of information on the histologic brain tumor subtypes, the most common being glioma, meningioma, and schwannoma. Race/ethnicity affects the incidence of different histologies of brain neoplasia^61^, further complicating identification of exposure-associated risk factors. Although it has been hypothesized that the risk of CNS tumors after IR exposure may vary by histologic subtype^3^, only Brenner et al.^14^ reported risks by histologic subtypes of CNS tumors among the atomic bomb survivors. Other studies analyzing mortality linked to brain tumors have no histologic information. Besides, the definition of this outcome was heterogeneous across studies (e.g., either limited to brain tumors or including tumors of the nervous system (not only central), either including benign tumors or limited to malignant tumors, etc.) which made it difficult to pool the studies together. However, most of them were based on national death registers on which the causes of death were coded according to the International Classification of Diseases, which ensures a certain reliability in the classification of deaths.

One major limitation of the considered studies was related to the over-representation of men in the different occupational cohorts, while biological responses to IR have been shown to differ by gender^62^. It has been suggested that susceptibility to IR-induced cancer is higher in women than in men^62^. In addition, incidence of CNS tumors in adults varies by sex, with a complex relationship depending on tumor biology (malignant vs. benign) and subtype^61^. Several studies included in our work were limited to men only^32–34,38–41^, while the others included both men and women. Only six studies carried out analyses for men and women separately^14,17,28,35–37^. However, women groups were much smaller compared to men groups, resulting in a low number of cancer cases and thus preventing robust results and conclusions. Furthermore, annual occupational radiation doses in women were shown to be substantially lower than those in men^18^, thus reinforcing the need to account for gender in the analyses. Nevertheless, studies of occupationally exposed medical workers^17,36,37^, as well as studies of cohorts exposed environmentally^42^, provide an opportunity to include a higher proportion of women in the analyses.

Despite the limitations described above related to specific study designs, the meta-analysis conformed to the PRISMA guidelines, ensuring a systematic and objective data analysis. The quality score between 7 and 9 on the Newcastle Ottawa Scale for all studies included in this review allowed to obtain a good homogeneity between the studies considered, confirmed by statistical tests. While, the NOS scale is the most accepted and used tool in systematic review work for quality studies assessment, some other scales have been proposed specifically in the field of radiation epidemiology to take into account the quality of the dose reconstruction^63^. However, as we exclude studies without dose assessment, the NOS scale seems appropriate to evaluate the quality of the studies.

Furthermore, to increase the robustness of our analysis and to account for unavoidable inherent heterogeneity between studies (mostly related to differences in populations, various types of radiation exposure, chronically or acute exposure), we used random effects models to calculate our estimates. Sensitivity analyses in which each pooled estimate (ERR) was calculated excluding each study one at a time and each group (e.g., flight attendants, nuclear workers or uranium miners, medical workers, etc.) revealed no substantial alteration of the overall heterogeneity. Then, our results can be considered as a good summary of the available literature on the risk of brain/CNS tumors after adult exposure to IR. The review of the studies also provided a better assessment of key points for future research in this area.

## Conclusion

This systematic review examined the relationship between low-to-moderate doses of IR when exposure occurred during adulthood/young adulthood and the risk of CNS tumors. There is no evidence of a dose–response association between IR exposure and risk of CNS tumors. Limitations of the studies include the lack of histological information on CNS tumors and large uncertainties in dose assessment. Further studies, ideally large-scale studies with adequate dosimetry and available information on potential confounding factors, will be essential to expand our knowledge on the effects of low-to-moderate doses of IR in adulthood/young adulthood on the CNS.

## Supplementary Information

Supplementary Information.

## Data Availability

All data generated or analyzed during this study are included in this published article (and its Supplementary Information files).
