# Supplementary material for: Ionizing radiation exposure during adulthood and risk of developing central nervous system tumors: systematic review and meta-analysis
Source: Sci Rep. 2022 Sep 28;12:16209. doi: 10.1038/s41598-022-20462-7 (PMC9519546; doi:10.1038/s41598-022-20462-7)
Supplement: Supplementary file 1 — Supplementary Information. [file 41598_2022_20462_MOESM1_ESM.docx]

# **Ionizing radiation exposure during adulthood and risk of developing central nervous system tumors: systematic review and meta-analysis**

Julie Lopes^1*^, Clémence Baudin^1^, Klervi Leuraud^1^, Dmitry Klokov^2^, Marie-Odile Bernier^1^

^1^Laboratory of Epidemiology (LEPID) – Institute for Radiological Protection and Nuclear Safety (IRSN), 92262 Fontenay-aux-Roses, France.

^2^Laboratory of Radiobiology and Radiotoxicology (LRTOX) – Institute for Radiological Protection and Nuclear Safety (IRSN), 92262 Fontenay-aux-Roses, France.

## **Supplementary data**

Table S1: Preferred Reporting Items for Systematic Reviews and Meta-analyses (PRISMA) 2020 checklist

| **Section and Topic** | **Item #** | **Checklist item** | **Reported on page** |
| --- | --- | --- | --- |
| **TITLE** | | |  |
| Title | 1 | Identify the report as a systematic review. | 1 |
| **ABSTRACT** | | |  |
| Abstract | 2 | See the PRISMA 2020 for Abstracts checklist. | 1 |
| **INTRODUCTION** | | |  |
| Rationale | 3 | Describe the rationale for the review in the context of existing knowledge. | 1-2 |
| Objectives | 4 | Provide an explicit statement of the objective(s) or question(s) the review addresses. | 2 |
| **METHODS** | | |  |
| Eligibility criteria | 5 | Specify the inclusion and exclusion criteria for the review and how studies were grouped for the syntheses. | 2 |
| Information sources | 6 | Specify all databases, registers, websites, organisations, reference lists and other sources searched or consulted to identify studies. Specify the date when each source was last searched or consulted. | 2; Figure 1 |
| Search strategy | 7 | Present the full search strategies for all databases, registers and websites, including any filters and limits used. | 2; Figure 1 |
| Selection process | 8 | Specify the methods used to decide whether a study met the inclusion criteria of the review, including how many reviewers screened each record and each report retrieved, whether they worked independently, and if applicable, details of automation tools used in the process. | 2 |
| Data collection process | 9 | Specify the methods used to collect data from reports, including how many reviewers collected data from each report, whether they worked independently, any processes for obtaining or confirming data from study investigators, and if applicable, details of automation tools used in the process. | 2-3 |
| Data items | 10a | List and define all outcomes for which data were sought. Specify whether all results that were compatible with each outcome domain in each study were sought (e.g. for all measures, time points, analyses), and if not, the methods used to decide which results to collect. | 2-3 |
|  | 10b | List and define all other variables for which data were sought (e.g. participant and intervention characteristics, funding sources). Describe any assumptions made about any missing or unclear information. | 2-3 |
| Study risk of bias assessment | 11 | Specify the methods used to assess risk of bias in the included studies, including details of the tool(s) used, how many reviewers assessed each study and whether they worked independently, and if applicable, details of automation tools used in the process. | 2 |
| Effect measures | 12 | Specify for each outcome the effect measure(s) (e.g. risk ratio, mean difference) used in the synthesis or presentation of results. | 3 |
| Synthesis methods | 13a | Describe the processes used to decide which studies were eligible for each synthesis (e.g. tabulating the study intervention characteristics and comparing against the planned groups for each synthesis (item #5)). | 3 |
|  | 13b | Describe any methods required to prepare the data for presentation or synthesis, such as handling of missing summary statistics, or data conversions. | 3 |
|  | 13c | Describe any methods used to tabulate or visually display results of individual studies and syntheses. | 3 |
|  | 13d | Describe any methods used to synthesize results and provide a rationale for the choice(s). If meta-analysis was performed, describe the model(s), method(s) to identify the presence and extent of statistical heterogeneity, and software package(s) used. | 3 |
|  | 13e | Describe any methods used to explore possible causes of heterogeneity among study results (e.g. subgroup analysis, meta-regression). | 3 |
|  | 13f | Describe any sensitivity analyses conducted to assess robustness of the synthesized results. | 3 |
| Reporting bias assessment | 14 | Describe any methods used to assess risk of bias due to missing results in a synthesis (arising from reporting biases). | 3 |
| Certainty assessment | 15 | Describe any methods used to assess certainty (or confidence) in the body of evidence for an outcome. | 3 |
| **RESULTS** | | |  |
| Study selection | 16a | Describe the results of the search and selection process, from the number of records identified in the search to the number of studies included in the review, ideally using a flow diagram. | 3; 6; Figure 1 |
|  | 16b | Cite studies that might appear to meet the inclusion criteria, but which were excluded, and explain why they were excluded. | NA |
| Study characteristics | 17 | Cite each included study and present its characteristics. | 6-7; Table 1 |
| Risk of bias in studies | 18 | Present assessments of risk of bias for each included study. | 6-7 |
| Results of individual studies | 19 | For all outcomes, present, for each study: (a) summary statistics for each group (where appropriate) and (b) an effect estimate and its precision (e.g. confidence/credible interval), ideally using structured tables or plots. | 6-7; Table 1 |
| Results of syntheses | 20a | For each synthesis, briefly summarise the characteristics and risk of bias among contributing studies. | 6-7 |
|  | 20b | Present results of all statistical syntheses conducted. If meta-analysis was done, present for each the summary estimate and its precision (e.g. confidence/credible interval) and measures of statistical heterogeneity. If comparing groups, describe the direction of the effect. | 7; Figure 2 |
|  | 20c | Present results of all investigations of possible causes of heterogeneity among study results. | 7 |
|  | 20d | Present results of all sensitivity analyses conducted to assess the robustness of the synthesized results. | NA |
| Reporting biases | 21 | Present assessments of risk of bias due to missing results (arising from reporting biases) for each synthesis assessed. | 7 |
| Certainty of evidence | 22 | Present assessments of certainty (or confidence) in the body of evidence for each outcome assessed. | 7 |
| **DISCUSSION** | | |  |
| Discussion | 23a | Provide a general interpretation of the results in the context of other evidence. | 7-8 |
|  | 23b | Discuss any limitations of the evidence included in the review. | 7-8 |
|  | 23c | Discuss any limitations of the review processes used. | 7-8 |
|  | 23d | Discuss implications of the results for practice, policy, and future research. | 7-8-9 |
| **OTHER INFORMATION** | | |  |
| Registration and protocol | 24a | Provide registration information for the review, including register name and registration number, or state that the review was not registered. | 2 |
|  | 24b | Indicate where the review protocol can be accessed, or state that a protocol was not prepared. | 2 |
|  | 24c | Describe and explain any amendments to information provided at registration or in the protocol. | NA |
| Support | 25 | Describe sources of financial or non-financial support for the review, and the role of the funders or sponsors in the review. | NA |
| Competing interests | 26 | Declare any competing interests of review authors. | 10 |
| Availability of data, code and other materials | 27 | Report which of the following are publicly available and where they can be found: template data collection forms; data extracted from included studies; data used for all analyses; analytic code; any other materials used in the review. | NA |

Table S2: List of the diseases studied and corresponding codes according to the International Classification of Diseases (ICD)

|  | **Diseases** |
| --- | --- |
| **ICD 7 version** |  |
| 193 | Malignant neoplasm of brain and other parts of nervous system |
| **ICD 9 version** |  |
| 191 | Malignant neoplasm of brain |
| 192 | Malignant neoplasm of other and unspecified parts of nervous system |
| 192.0 | Cranial nerves |
| 192.1 | Cerebral meninges |
| 225 | Benign neoplasm of brain and other parts of nervous system |
| **ICD 10 version** |  |
| C70 | Malignant neoplasm of cerebral meninges |
| C70.0 | Cerebral meninges |
| C70.1 | Spinal meninges |
| C70.9 | Meninges, unspecified |
| C71 | Malignant neoplasm of brain |
| C72 | Malignant neoplasm of spinal cord, cranial nerves and other parts of central nervous system |
| C72.0 | Spinal cord |
| C72.2 | Olfactory nerve |
| C72.5 | Other and unspecified cranial nerves |
| C72.9 | Central nervous system, unspecified |
| C75.1 | Pituitary gland |
| C75.3 | Pineal gland |
| D32 | Benign neoplasm of meninges |
| D33 | Benign neoplasm of brain and other parts of central nervous system |
| D33.0 | Brain, supratentorial |
| D33.2 | Brain, unspecified |
| D42 | Neoplasm of uncertain or unknown behavior of cerebral meninges |
| D43 | Neoplasm of uncertain or unknown behavior of brain and central nervous system |
| D43.0 | Brain, supratentorial |
| D43.2 | Brain, unspecified |

Table S3: Morbidity and/or mortality data of studies reporting SIR/SMR values in the retrieved studies

| **First author, year** | **Country** | **Population** | **Design** | **Exposure assessment** | **Outcome(s)** | **Major results** | **NOS scores** |
| --- | --- | --- | --- | --- | --- | --- | --- |
| **Nuclear workers and uranium miners** | | | | | | | |
| Boice et al. 2022 | USA | 130,773 (M), 4,420 (F) nuclear power plant workers | Cohort | Mean dose to the brain: 33.2 mGy (max: 0.83 Gy) | Brain, central nervous system cancer (ICD-9: 191-192) | SMR (95% CI): 0.90 (0.80, 1.02), n_deaths_= 274 | 8 |
| Boice et al. 2021 | USA | 19,808 (M), 6,520 (F) workers at the Los Alamos National Laboratory | Cohort | Brain radiation absorbed dose, combining external and internal sources for Pu: mean: 11.6 mGy, median: 0.76 mGy, max: 760 mGy | Brain, central nervous system cancer (ICD-9: 191-192) | SMR (95% CI): 0.79 (0.64, 0.97), n_deaths_= 94 | 8 |
| Kreuzer et al. 2021 | Germany | 35,204 (M) underground uranium miners | Cohort | Mean cumulative exposure to radon: 364 WLM and silica dust: 7.6 mg/m^3^-years | Brain, central nervous system cancer (ICD-10: C70-C72) | SMR (95% CI): 0.83 (0.67, 1.02), n_deaths_= 90 | 7 |
| Kelly-Reif et al. 2019 | Czech Republic | 16,434 (M) underground uranium miners | Cohort | Cumulative radon exposure: 53 WLM (1.2-1,121.9 WLM) | Brain cancer (ICD-9: 191) | SMR (95% CI): 0.76 (0.35, 1.18), n_deaths_= 13  SIR (95% CI): 0.83 (0.38, 1.29), n_diseases_= 13 | 7 |
| Golden et al. 2019 | USA | 2,514 (M) Mallinckrodt uranium processing workers | Cohort | Brain dose from all sources of external and internal radiation combined: mean 37.2 mGy; max: 750 mGy | Brain, central nervous system cancer (ICD-9: 191-192) | SMR (95% CI): 1.85 (1.16, 2.80), n_deaths_= 22 | 8 |
| Rage et al. 2017 | France | 5,400 (M) uranium miners | Cohort | Cumulative exposure (WLM), mean (se): 35.1 (69.9), median (min-max): 10.8 (0.002-960.1) | Brain, central nervous system tumor (ICD-10: C70-C72, D32-D33, D42-D43) | SMR (95% CI): 1.43 (0.95, 2.07), n_deaths_= 28 | 7 |
| Navaranjan et al. 2016 | Canada | 28,546 (M), 413 (F) uranium miners | Cohort | Cumulative radon exposure: mean: 21.0 WLM, range: 0.0-875.1 WLM (M); mean: 0.2 WLM, range: 0.0-16.3 WLM (F) | Brain, central nervous system cancer (ICD-9: 191-192) | SMR (95% CI): 0.80 (0.62, 1.02), n_deaths_= 67  SIR (95% CI): 0.78 (0.60, 0.98), n_diseases_= 70 | 8 |
| Boice et al. 2014 | USA | 4,004 (M), 973 (F) mound workers | Cohort | Mean dose from external radiation: 26.1 mSv (max: 939.1 mSv). Mean lung dose from internal exposure: 100.1 mSv (max: 17.5 Sv). Mean liver dose from external and internal radiation: 34.6 mSv (max: 2.3 Sv) | Brain, central nervous system cancer (ICD-9: 191-192) | SMR (95% CI): 0.67 (0.35, 1.17), n_deaths_= 12 | 8 |
| Boice et al. 2011 | USA | 5,335 (M), 466 (F) nuclear workers at Rocketdyne | Cohort | Mean dose from external radiation: 13.5 mSv (max: 1 Sv) and the mean lung dose from external and internal radiation combined: 19.0 mSv (max: 3.6 Sv) | Brain, central nervous system cancer (ICD-9: 191-192) | SMR (95% CI): 1.21 (0.78, 1.78), n_deaths_= 25 | 8 |
| Howe et al. 2004 | USA | 47,311 (M), 6,387 (F) nuclear power industry workers | Cohort | Mean cumulative equivalent dose: 28.5 mSv (M), 4.6 mSv (F) and 25.7 mSv for the all cohort | Brain, central nervous system cancer (ICD: NA) | SMR (95% CI): 0.85 (0.54, 1.28), n_deaths_= 23 | 8 |
| Iwasaki et al. 2003 | Japan | 176,000 (M) nuclear industry workers | Cohort | Mean cumulative dose per person in the total study population: 12.0 mSv | Neoplasm of malignant, benign and unspecified nature of brain and central nervous system (ICD: NA) | SMR (95% CI): 0.69 (0.45, 1.01), n_deaths_= 26 | 8 |
| **Medical workers** | | | | | | | |
| Boice et al. 2021 | USA | 55,218 (M), 53,801 (F) medical and associated radiation workers | Cohort | Mean cumulative absorbed dose to the brain: 18.9 mGy (max: 1.08 Gy) | Brain, central nervous system cancer (ICD-9: 191, 192.0-192.1) | SMR (95% CI): 0.89 (0.76, 1.04), n_deaths_= 167 | 8 |
| Lee et al. 2021 | South Korea | 53,582 (M), 40,338 (F) diagnostic medical radiation workers | Cohort | Mean cumulative badge dose: 7.20 mSv (IQR: 0.21-5.41 mSv) | Brain, central nervous system cancer (ICD-10: C70-C72) | SIR (95% CI): 1.45 (0.94, 2.24), n_diseases_= 43 (M/F)  SIR (95% CI): 1.04 (0.72, 1.52), n_diseases_= 27 (M)  SIR (95% CI): 1.61 (0.98, 2.62), n_diseases_= 16 (F) | 8 |
| Zielinski et al. 2009 | Canada | 23,580 (M), 43,982 (F) medical radiation workers | Cohort | Cumulative lifetime dose: 3.78 mSv | Brain, central nervous system cancer (ICD: NA) | SMR (90% CI): 0.61 (0.40, 0.90), n_deaths_= 19 (M/F); SMR (90% CI): 0.68 (0.39, 1.11), n_deaths_= 12 (M); SMR (90% CI) 0.52 (0.25, 0.98), n_deaths_= 7 (F)  SIR (90% CI): 0.72 (0.52, 0.97), n_diseases_= 32 (M/F); SIR (90% CI): 0.95 (0.64, 1.36), n_diseases_= 22 (M); SIR (90% CI): 0.47 (0.26, 0.80), n_diseases_= 10 (F) | 8 |
| **Military using nuclear materials** | | | | | | |  |
| Tao et al. 2022 | USA | 145,023 (M) shipyard radiation workers | Cohort | Mean lifetime dose: 15.4 mSv, median: 0.82 mSv. | 1 - Brain, central nervous system cancer (ICD: NA)  2 - Benign neoplasm of the eye, brain, and other part (ICD: NA) | 1 - SMR (95% CI): 0.81 (0.73, 0.89), n_deaths_= 372  2 - SMR (95% CI): 0.90 (0.55, 1.38), n_deaths_= 20 | 8 |
| Gillies et al. 2022 | UK | 21,357 (M) UK participants in the UK's atmospheric nuclear weapons tests and experimental programs compared to a group of 22,312 (M) controls | Cohort | 8% of the total participant cohort had non-zero recorded radiation doses (mean dose from gamma radiation: 9.9 mSv) | 1 - Brain and central nervous system tumor (ICD-9: 191-192, 225 / ICD-10: C70-C72, D32-D33)  2 - Benign brain and central nervous system tumor (ICD-9: 225 / ICD-10: D32-D33) | 1 - SMR (95% CI): 1.02 (0.84, 1.22), n_deaths_= 116  1 - SIR (95% CI): 1.12 (0.95, 1.32), n_diseases_= 146  2 - SIR (95% CI): 2.08 (1.50, 2.80), n_diseases_= 43 | 8 |
| Boice et al. 2020 | USA | 114,270 (M) military participants at eight aboveground nuclear weapons test series | Cohort | Gamma radiation dose: mean: 6 mSv, max: 908 mSv | Brain, central nervous system cancer (ICD-9: 191-192) | SMR (95% CI): 0.94 (0.86, 1.03), n_deaths_= 495 | 7 |
| **Flight attendants** | | | | | | | |
| Dreger et al. 2020 | Germany | 6,006 (M) cockpit crew, 17,017 (F) cabin crew | Cohort | Collective cumulative effective doses (in mSv): 44.1 (IQR: 30.5-54.1, max: 99.7) and 25.1 (IQR: 10.5-46.6, max: 96.7) for male cockpit and female cabin crew respectively | Brain, central nervous system cancer (ICD: NA) | SMR_c_ (95% CI): 2.01 (1.15, 3.28), n_deaths_= 23 (M); SMR_c_ (95% CI): 1.26 (0.60, 2.36), n_deaths_= 14 (F) | 8 |
| Yong et al. 2014 | USA | 5,958 (M), 6 (F) cockpit crew | Cohort | Mean annual cosmic radiation dose: 1.4 mSv (median: 1.4 mSv, range: 0.0042-2.8 mSv) | Brain, central nervous system cancer (ICD: NA) | SMR (95% CI): 1.39 (0.95, 1.96), n_deaths_= 32 | 8 |
| Pinkerton et al. 2012 | USA | 1,701 (M), 9,610 (F) flight attendants | Cohort | Median estimated cumulative radiation dose: 12.7 mSv (range: 0.33-102 mSv) | Brain, central nervous system cancer (ICD: NA) | SMR (95% CI): 1.03 (0.56, 1.73), n_deaths_= 14 (M/F)  SMR (95% CI): 1.97 (0.72, 4.30), n_deaths_= 6 (M)  SMR (95% CI): 0.76 (0.33, 1.49), n_deaths_= 8 (F) | 7 |
| Pukkala et al. 2012 | Finland, Iceland, Norway, Sweden | 1,559 (M), 8,507 (F) airline cabin crew | Pooled cohort | Estimated cosmic radiation dose according to the percentage of cabin crew members: <5 mSv: 30%; 5-14.9 mSv: 34%; 15-34.9 mSv: 29%; ≥35 mSv: 6%. | Brain, central nervous system cancer (ICD-7: 193) | SIR (95% CI): 0.92 (0.60, 1.34), n_diseases_= 26 (M/F)  SIR (95% CI): 1.28 (0.47, 2.79), n_diseases_= 6 (M)  SIR (95% CI): 0.85 (0.52, 1.31), n_diseases_= 20 (F) | 7 |
| Pukkala et al. 2003 | Denmark, Finland, Iceland, Norway, Sweden | 10,051 (M) airline pilots | Pooled cohort | Estimated dose according to the percentage of airline pilots: 1-2,999 µSv: 46%; 3,000-9,999 µSv: 15%; 10,000-19,999 µSv: 14%; ≥20,000 µSv: 11%. | Brain, central nervous system cancer (ICD: NA) | SIR (95% CI): 0.84 (0.50, 1.33), n_diseases_= 18 | 7 |
| **Chernobyl cleanup workers** | | | | | | | |
| Rahu et al. 2013 | Estonia, Latvia, and Lithuania | 17,040 Chernobyl cleanup workers (M) | Cohort | External whole-body radiation dose: average dose: 10.9 cGy (9.9 cGy, 11.8 cGy and 10.9 cGy in Estonian, Latvian, and Lithuanian sub-cohorts respectively) and interquartile range of 5.2–16.3 cGy | 1 – Brain, central nervous system cancer (ICD-10: C70-C72)  2 – Brain cancer (ICD-10: C71) | 1 – SIR (95% CI): 1.34 (0.92, 1.89), n_diseases_= 32  2 – SIR (95% CI): 1.25 (0.83, 1.81), n_diseases_= 28 | 8 |

Figure S1: Standardized mortality ratio (SMR) and 95% confidence interval (CI) for brain/CNS tumors death in IR exposed populations compared with general populations as reference.

SMR_pooled_ was calculated using both the natural logarithms of the SMRs and their standard errors for each included study. The standard error of log-SMR was estimated by $ln(\frac{\left( \frac{SMR upper CI}{SMR lower Ci} \right)}{3.92})$. The log-SMRs were then pooled together to generate and plot an overall effect size using the DerSimonian–Laird random-effect method. Lastly, the pooled log-SMRs were exponentiated as a back-transformation for interpretation. When only observed (D) and expected (E) numbers of deaths were reported in the studies, the SMR and associated lower and upper 95% CI-bounds were calculated as follows: SMR=D/E, SMR_L_=$SMR(1-{\frac{Z\alpha}{{2D}^{1/2}})}^{2}$, and SMR_U_=SMR× ($\frac{D+1}{D}$) × (1+${\frac{Z\alpha}{{2(D+1)}^{1/2}})}^{2}$, where Zα is the 100(1-α/2) percentile of the unit normal distribution and α=0.05.

Figure S2: Standardized incidence ratio (SIR) and 95% confidence interval (CI) for brain/CNS tumors morbidity in IR exposed populations compared with general population as reference.

The same statistical method as for the calculation of the SMR was performed.
